# A Case of Profuse Hæmaturia, the Result, of Injury Treated Successfully with Gallic Acid

**Published:** 1847-07

**Authors:** James S. Hughes

**Affiliations:** Surgeon to Jervis-Street Hospital


*A Case of profuse Hæmaturia, the result of Injury, treated successfully with Gallic Acid.* By JAMES S. HUGHES, F. R. C. S., Surgeon to Jervis-Street Hospital.—John Hyland, aged 30, a Custom House porter, admitted into hospital on September 8th, 1846; states that, about half an hour before admission, he was employed in lowering a cask full of sugar, when he was struck by the handle of the windlass with great violence in the left lumbar region; he was rendered insensible for a short time, and was carried into the hospital. On examination an extensive ecchymosis was found to exist along the left side of the spinal column and lumbar region: the ninth, tenth, and eleventh ribs were fractured close to the vertebræ; there was excruciating pain on pressure over the region of the kidney; abdomen tympanitic; testicles retracted; countenance deadly pale, covered with cold perspiration, and highly expressive of pain; pulse quick and feeble. Soon after admission he expressed a desire to make water, and expelled, with much difficulty, more than half a pint of pure blood. Ordered six leeches to the seat of pain, and to have a draught containing acetate of lead and acetum opii every second hour.

9th. Slept very badly; pain in the left lumbar region intense; much increased by the slightest pressure: urine highly loaded with blood; finds considerable difficulty in emptying his bladder; bowels freed during the night; the leeches were repeated, and the draughts continued.

10th. Pain somewhat relieved by the leeches; made several attempts to pass his urine during the night, but could not do so; constant desire to pass water; bladder distended. A gum elastic catheter having been passed into the bladder, a considerable quantity of blood and urine were drawn off; there were several long clots of blood discharged. My colleague, Doctor Neligan, having suggested to me a trial of gallic acid, I was induced to order it in the form of pills, with extract of gentian, two grains and a half of the acid in each pill, which were taken at intervals of three hours.

11th. Considerably improved; the quantity of blood in the urine much diminished; after the third pill, the presence of gallic acid in the urine was detected by the addition of a few drops of tinctura ferri, sesq. chlorid., which converted it into a perfect ink. The pills were repeated.

12th. Passed a good night; made water freely; urine limpid, quite devoid of blood; pain in the lumbar region considerably decreased; pulse sixty-four, soft. Discharged cured on the 18th.

Gallic acid has proved a most useful addition to our list of astringents. Both as an external and an internal remedy in hæmorrhages its character stands high, and justly so; it is now generally alleged to be the active principle in Ruspini's celebrated Styptic, which Dr. Thompson is of opinion consists of gallic acid, sulphate of zinc, opium, alcohol, and rose water; the gallic acid evidently being the active ingredient. Sometime since I saw the power of Ruspini's Styptic put to the test in the case of a gentleman who had some of the branches of the palmar arch of arteries opened by the bursting of a bottle of soda water; profuse hæmorrhage having ensued, and attempts to secure the bleeding vessels having been tried in vain, graduated pressure was applied, but to such an extent, and for such a length of time, that sloughing of the palm of the hand ensued, with inflammation extending up the forearm, and considerable fever, together with repeated periodical hæmorrhages, by which the patient was considerably reduced: at this stage I saw the case in consultation, when it was agreed to give a trial to this powerful styptic, and a single application of it was followed by an immediate arrest of the hæmorrhage, and recovery. As a local application in aphthous ulceration of the mouth and tongue, I can speak highly of gallic acid ; it is also a valuable injection in the gleety stage of gonorrhœa. As an internal remedy, gallic acid has been used with great success by Dr. Simpson and others in certain forms of uterine hæmorrhage, and with this advantage over most other anti-hæmorrhagic medicines, that it had no constipating effect on the bowels ; but as gallic acid passes directly to the kidneys, acting thereby as a direct astringent, the urine becoming impregnated with it very soon after its exhibition, it consequently is an astringent peculiarly suited to hæmorrhages from the urinary organs, and as such has been strongly recommended by Drs. Steevenson, Golding Bird, and others. Dr. Steevenson has published in the Edinburgh Medical and Surgical Journal, the following case of obstinate hæmaturia, successfully treated by gallic acid. The patient was a boy fourteen years of age, who had been passing blood with his urine for several months, supposed to have been caused by a blow which he had received in the lower abdomen from one of his school-fellows. After ineffectual attempts to arrest the discharge of blood, three grains of gallic acid were given every three hours for four days, when the discharge subsided, and did not return. In the case which I have brought forward, we found that the large doses of acetate of lead, combined with opium, did not check the hæmorrhage ; whereas the bleeding ceased altogether after the exhibtion of the third dose of the gallic acid, at which time the presence of the acid in the urine was proved by the addition of the tinct. ferri sesq. chlorid. —*Ibid.*

